# An obscure cause of bowel obstruction: Jejunal herniation into uterine cavity

**DOI:** 10.1016/j.ijscr.2023.108806

**Published:** 2023-09-09

**Authors:** Salman Idrees, Alessandro Bellomo, Thifhelimbilu Luvhengo

**Affiliations:** a University of the Witwatersrand, South Africa; b Charlotte Maxeke Johannesburg Academic Hospital, South Africa

**Keywords:** Internal hernia, Manual vacuum aspiration, Small bowel obstruction, Uterine perforation

## Abstract

**Introduction and importance:**

Adhesions and external hernias are the two most common causes of small bowel obstruction. Perforation of organs within the abdomen or pelvis following manual vacuum aspiration is known to lead to an acute presentation.

**Case presentation:**

We report a case of a 33-year-old female with small bowel obstruction due to herniation of a loop of intestine through a uterine defect with symptoms starting 63 days following manual vacuum aspiration.

**Clinical discussion:**

Intra-abdominal or pelvic perforations usually present immediately which makes our case unique as the patient started having symptoms 63 days post manual vacuum aspiration. The most feared complication of prolonged small bowel obstruction is ischaemia which may lead to perforation.

In our case, it is plausible that jejunum partially herniated into the uterine cavity shortly after manual vacuum aspiration, forming a jejunal plug, leading to the delayed onset of symptoms. This delay in onset of symptoms might have led to progressive massive dilatation of the small bowel and subsequent ischaemic necrosis.

**Conclusion:**

Detailed history taking is pertinent as bowel obstruction could still occur a prolonged period after manual vacuum aspiration. A double-contrast enhanced CT scan of the abdomen proves invaluable in the context of surgical planning and facilitating the collaboration of a multidisciplinary team, particularly when the underlying causes of bowel obstruction remain elusive upon initial presentation.

## Highlights

• “The risk of uterine perforation as a complication of MVA is 0.06 to 0.36%.”
• “2.4% incidence of SBO post hysterectomy.”
• “She had a history of having had a MVA performed for an incomplete miscarriage 63 days prior to the start of her symptoms.”
• “Contrast enhanced CT of the abdomen showed features of SBO with a transition point in the pelvis.”
• “There was jejunal herniation into the uterine cavity”
• “Intra-abdominal or pelvic perforations usually present immediately which makes our case unique as the patient started having symptoms 63 days post MVA.”

## 1 Background

Small bowel obstruction (SBO) is a common surgical emergency which needs to be diagnosed and treated timeously as it can become life-threatening. The most common causes of SBO are adhesions due to previous abdominal surgery, followed by hernias. Rare causes include tumours, intussusception, gastrointestinal tuberculosis (GI TB) [11] and internal hernias. Amongst the most dreaded complications is strangulation, which may lead to ischaemia, necrosis and perforation [1,2].

Manual vacuum aspiration (MVA) is a safe surgical method of uterine evacuation for the removal of retained products of conception in a patient with an incomplete miscarriage. Fewer complications are reported for MVA compared to curettage. Although it is safe and effective, rare uterine perforations can occur and can be life-threatening, especially with intra-abdominal organ injury [3,4,7,8].

The risk of uterine perforation as a complication of MVA is 0.06 to 0.36 %. Close to 50 % of these are due to the suction cannula and mostly occurring at the fundus and anterior wall [3,4]. Majority of these cases involve multiparous women [4]. Arabkhazaeli et al., found a 2.4 % incidence of SBO post hysterectomy [13]. Uterine perforations present as an emergency requiring urgent treatment and may be associated with life-threatening complications like intra-abdominal haemorrhage, hollow viscus perforations including the gastro-intestinal tract (GIT) and bladder [[3], [4], [5],^7^,^8^].

## 2 Methods

Retrospective report from the patient's file reported in line with the SCARE criteria [10].

### 2.1 Consent

Written informed consent was obtained from the patient for publication of this case report and accompanying images. A copy of the written consent is available for review by the Editor-in-Chief of this journal on request.

### 2.2 Ethics approval

Ethics approval for this study was granted by the Human Research Ethics Committee (Medical) at the third floor, Faculty of Health Sciences, Phillip Tobias Building, 29 Princess of Wales Terrace, Parktown, Johannesburg, South Africa, 2193. The clearance certificate number is M230457 and was granted on the 14th of July 2023.

## 3 Case presentation

A 33-year-old black-African female, G1P0M1 with no co-morbidities, presented to a level 2 hospital with a one-week history of obstipation, vomiting and abdominal distension associated with abdominal pain. The pain radiated to the umbilicus and suprapubic regions. She had a history of having had a MVA performed for an incomplete miscarriage, 63 day prior to the start of her symptoms. She had no previous history of abdominal surgery, and no external hernias were found. She was then referred to our facility, a level 3 hospital for further review and management.

On assessment, she was alert but generally wasted, with no evidence of dehydration. She was normotensive with a blood pressure of 125/75 mmHg but had a tachycardia of 127 bpm. Her abdomen was distended with peritonitis and bowel sounds were absent. Her rectum was empty with an impression of a mass anteriorly. Arterial blood gas showed a pH of 7.516 and HCO_3_^−^ of 31.8 mmol/L (see Table 1). Her white cell count (WCC) and CRP were elevated at 26.64 × 10(9)/L and 233 mg/L respectively. The patient was initially resuscitated with intravenous fluids and electrolyte abnormalities were corrected before she was sent for radiological investigations.

**Table 1:** Laboratory results during admission.

Parameters	Result	Laboratory reference values
pH	7.516	7.35–7.45
HCO3	31.8 mmol/L	22–28
pCO2	39.2 mmol/L	35–45
Base excess	8.1 mmol/L	−2–2
Lactate	1.8 mmol/L	< 2
Potassium	2.9 mmol/L	3.6–5.2
Sodium	126 mmol/L	135–145
WCC	26.64 × 10^9^/L	4.5–11 × 10^9^/L
CRP	233 mg/L	<10

**Fig. 1:**
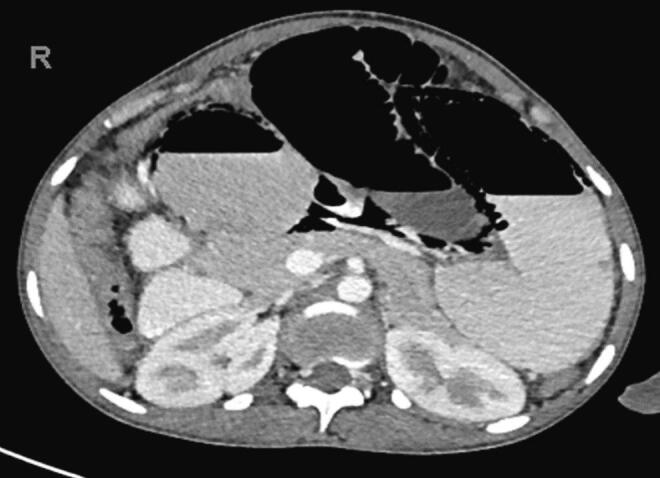
Contrast enhanced CT abdomen showing endometrial free fluid with poor myometrial enhancement. There is also a pelvic transition point.

**Fig. 2:**
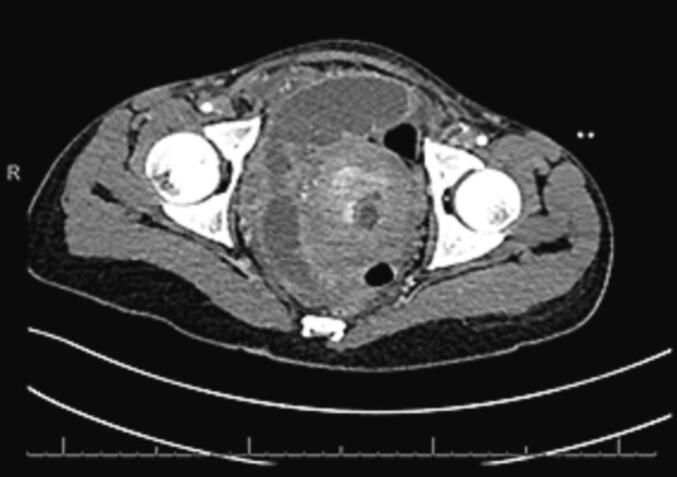
Contrast enhanced CT abdomen showing dilated loops of proximal small bowel with air-fluid levels and pneumatosis intestinalis.

Plain erect abdominal radiograph showed dilated loops of small bowel with multiple air-fluid levels. Contrast enhanced CT of the abdomen showed features of SBO with a transition point in the pelvis. Her uterus was large, bulky and inhomogeneous with endometrial enhancement. The CT scan also showed endometrial free fluid with poor myometrial enhancement (Fig. 1). The dilated loops of small bowel had pneumatosis intestinalis (Fig. 2). Both the superior mesenteric artery and vein were normal. Differential diagnoses were pelvic inflammatory disease with adhesions, ileo-caecal tuberculosis or internal herniation of small bowel.

**Fig. 3:**
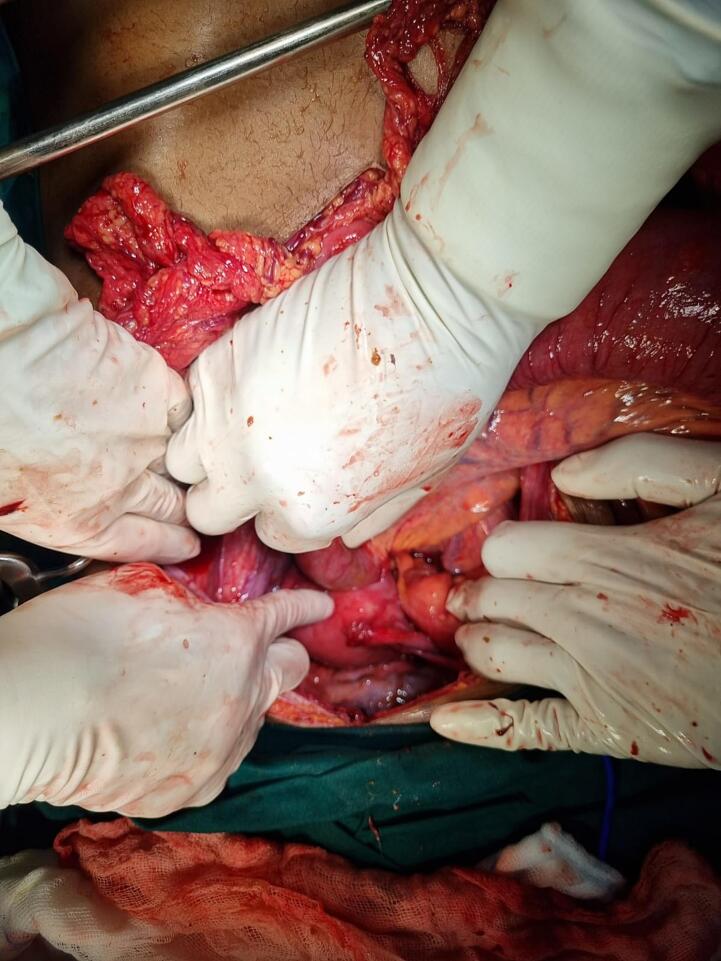
Intra-operative photo showing the jejunum herniated into the uterus.

The patient was taken to theatre for an exploratory laparotomy which revealed a 30 cm long segment of necrotic small bowel, 15 cm from the ligament of Treitz. There was jejunal herniation into the uterine cavity (Fig. 3). There was faecal contamination. The necrotic bowel was resected and the gynaecology team repaired the uterine defect. It was unsafe to do an anastomosis due to abdominal sepsis. The necrotic bowel was resected and a double barrel jejunostomy was fashioned.

The challenge with this patient was that the segment of bowel involved was very proximal which led to a high output proximal stoma. She received total parenteral nutrition (TPN), and her stoma losses were replaced. The stoma was then reversed 6 weeks after the surgery, and the patient was able to return to her usual activities.

## 4 Discussion

To our knowledge and extensive literature search, jejunal herniation into the uterus has never been documented as a cause of bowel obstruction. Intra-abdominal or pelvic perforations usually present immediately which makes our case unique as the patient started having symptoms 63 days post MVA. Detailed history taking is pertinent and this diagnosis could still be considered after a prolonged period. This case also highlights the importance of radiological imaging in patients who present with features of SBO with no obvious cause.

The common presenting symptoms of SBO are colicky abdominal pain and bilious vomiting. The dominance of either of the symptoms is dependent on whether the obstruction is proximal or distal [1,2]. On presentation SBO is categorised into simple or complicated. The most feared complication of prolonged SBO is ischaemia which may lead to perforation [1]. Patients presenting with complicated SBO need to be resuscitated and taken to theatre urgently. When the obstruction of small bowel is due to adhesions, initial attempt at a trial of conservative treatment is recommended unless patient has features of ischaemic bowel. Surgery preceded by imaging is also indicated where the cause of SBO is not evident such as in patients without a previous history of surgery or evidence of external hernias [1,2].

GI TB predominantly manifests at the ileocecal junction but can potentially affect any segment of the GI Tract. Furthermore, GI TB has been associated with the development of bowel obstruction [11]. Given the elevated prevalence of TB in South Africa [12] and the patient's wasted state, ileocecal TB was considered as a potential differential diagnosis in this case.

While small bowel herniation due to MVA associated uterine perforation has been described in the literature, usually these patients present in the acute setting [5,7]. The Onset of symptoms in our patient was 63 days post MVA. Her symptoms, clinical examination and subsequent imaging studies confirmed the diagnosis of bowel obstruction, warranting exploratory laparotomy. It is plausible that jejunum partially herniated into the uterine cavity shortly after MVA, forming a jejunal patch, leading to the delayed onset of symptoms. This delay in onset of symptoms might have led to progressive massive dilatation of the small bowel and subsequent ischaemic necrosis.

Patients presenting with SBO without an obvious cause should undergo double contrast enhanced CT scan of the abdomen before they are taken to theatre for exploration or discharged [9]. In this case imaging assisted in making an obscure diagnosis, better preparing a multidisciplinary surgical team in order to adequately manage the presenting pathology.

## 5 Conclusions

Detailed history taking is pertinent as bowel obstruction could still occur a prolonged period after MVA. A double-contrast enhanced CT scan of the abdomen proves invaluable in the context of surgical planning and facilitating the collaboration of a multidisciplinary team, particularly when the underlying causes of bowel obstruction remain elusive upon initial presentation.

## Consent

Written informed consent was obtained from the patient for publication and any accompanying images. A copy of the written consent is available for review by the Editor-in-Chief of this journal on request.

## Ethical approval

Ethics approval has been obtained from: Human research ethics committee (Medical), University of the Witwatersrand. (Wits - HREC) Third floor, Faculaty of Health Sciences, Phillip Tobias Building, 29 Princess of Wales Terrace, Parktown, 2193.

Clearance certificate number:M230457.

## Funding

No role of study sponsors.

## Author contribution

Muhammad Salman Idrees: Study concept and design, data collection, data interpretation and writing the paper.

Alessandro Bellomo: Data interpretation and editing.

Prof. Thifhelimbilu Luvhengo: Interpretation and editing.

## Guarantor

Muhammad Salman Idrees.

## Research registration number

This case report is not a first in man study.

## Conflict of interest statement

Nothing to declare.
